# MicroRNA-934 facilitates cell proliferation, migration, invasion and angiogenesis in colorectal cancer by targeting B-cell translocation gene 2

**DOI:** 10.1080/21655979.2021.1996505

**Published:** 2021-12-09

**Authors:** Bo Li, Xianyi Liu, Guogang Wu, Jiawen Liu, Shouliang Cai, Fuxin Wang, Chunyu Yang, Jisheng Liu

**Affiliations:** a Department of General Surgery, Ansteel Group General Hospital, Anshan, Liaoning, China; b Department of General Surgery, Affiliated Zhongshan Hospital of Dalian University, Dalian, Liaoning, China

**Keywords:** Angiogenesis, btg2, cell proliferation, cell migration, miR-934

## Abstract

Colorectal cancer (CRC) is a global public health issue with increasing prevalence. MicroRNA-934 (miR-934) is a kind of non-coding RNA involved in the regulation of diverse cancers. Though previous researches have revealed part of association between miR-934 and CRC, the role of miR-934 in CRC pathogenesis has not been completely explored yet. In this study, we aim to investigate the effect of miR-934 on cell proliferation, migration, invasion and angiogenesis in CRC. Accordingly, miR-934 was found to be over-expressed in SW480 and HCT116 cells, two typical CRC cell lines. Meanwhile, miR-934 knockdown significantly inhibited cell proliferation and induced cell cycle arrest in SW480 and HCT116 cells. It was further validated that miR-934 knockdown displayed an inhibitory effect on cell migration and invasion in SW480 and HCT116 cells. Additionally, miR-934 deficiency markedly decreased VEGF expression in SW480 and HCT116 cells and suppressed capability of CRC cells to promote tube formation in vascular endothelial cells, which suggests the pro-angiogenesis role of miR-934 in vitro. Dual luciferase reporter assay further showed that miR-934 directly bound to B-cell translocation gene 2 (BTG2). BTG2 knockdown reversed the inhibitory effect of miR-934 silencing on cell proliferation, migration, invasion, and angiogenesis in SW480 and HCT116 cells. In summary, this study suggests that miR-934 facilitates CRC progression by targeting BTG2, and further highlights the role of miR-934 in pathogenesis of CRC.

## Introduction

Colorectal cancer (CRC) is a common gastrointestinal disease occurring in colon and rectum[1]. It is estimated that approximately 0.9 million deaths are attributed to CRC annually [2], and it has been acknowledged as the second leading cause of cancer-related deaths [3]. The pathogenic factors of CRC are complicated, while it is strongly associated with unhealthy lifestyles, genetic and environmental factors[1]. Nowadays, tumor removal by surgery and chemotherapy are the most common therapeutic approaches for CRC. However, CRC patients always suffer from severe side effects and poor prognosis after these typical treatment [4]. Thus, it is necessary to further clarify the pathogenesis of CRC and find novel therapeutic strategy for CRC.

MicroRNAs (miRNAs) are a group of short non-coding RNAs with 19–22 nucleotides in length and single-strand structure. It is well established that miRNAs display an important role in regulating biological behaviors of tumor progression. Previous studies have determined that miR-934 functions as an oncogene in several kinds of malignancies [5–7]. For instance, its over-expression significantly promotes cell proliferation and suppresses cell apoptosis in ovarian cancer (OC) cells [6]. In addition, miR-934 is reported to be highly expressed in CRC patients [8], and exosomal miR-934 promotes the metastasis of CRC toward liver by inducing M2 type macrophage polarization [9]. Though previous researches have revealed part of association between miR-934 and CRC, the role of miR-934 in CRC pathogenesis has not been completely explored yet.

Tumor microenvironment (TME) refers to a cellular environment comprising tumor cells and their surrounding nonmalignant cells [10]. Of note, TME has been documented to play a crucial role in the regulation of tumor proliferation, angiogenesis, invasion, metastasis and chemo-resistance, which are well recognized as the hallmarks of cancer progression [11]. Besides, the aberrantly regulated miRNAs are reported to regulate these physiological progresses by affecting the interactions between malignant cells and nonmalignant cells [11]. Specially, interventations on the interaction between tumor cells and vascular epithelial cells have shown critical effects on the therapeutic management of cancer. Due to the high proliferative rate, tumor cells will induce elevated levels of pro-angiogenic factors to promote angiogenesis, which leads to the generation of a new vascular network [12]. Importantly, CRC is a typical malignancy that requires active angiogenesis [13], which suggests that tumor angiogenesis could be an important target for the therapy of CRC.

Accordingly, microRNAs usually function by binding to their corresponding mRNA to promote the degradation of target genes [14]. In this study, B-cell translocation gene 2 (BTG2) is predicted to be a potential target of miR-934 according to the bioinformatics database. Actually, BTG2 is a common anti-proliferative gene with decreased expression in several tumors, such as gastric cancer, breast carcinoma and colon cancer [15–18]. Besides, it is further documented that BTG2 participates in the regulation of angiogenesis in miR-27a-mediated development of pancreatic cancer [19]. In summary, our study hypothesizes that miR-934 might regulate cell proliferation, migration, invasion and angiogenesis of CRC by targeting at BTG2. Meanwhile, we aim to further clarify the pathogenesis of CRC and provide novel insights for the development of CRC therapeutic strategy.

## Materials and methods

### Cell culture

Human CRC cell lines (HT-29, LoVo, SW480 and HCT116) and human umbilical vein endothelial cells (HUVECs) were purchased from Procell Life Science &Technology (Wuhan, China). The normal Human Colonic Epithelial Cells were obtained from Zhong Qiao Xin Zhou Biotechnology (Shanghai, China). HT-29 and HCT116 cells were cultured in McCoy’s 5A medium (PM150710, Procell, Wuhan) with 10% fetal bovine serum (FBS, 04–011-1A, BI) in an incubator at 37°C in 5% CO_2._ SW480 cells were cultured in L-15 medium (PM151010, Procell, Wuhan) supplemented with 10% FBS in an incubator at 37°C in 5% CO_2°C_ LoVo cells were cultured in Ham’s F-12 K (PM150910, Procell, Wuhan) medium containing 10% FBS in an incubator at 37°C in 5% CO_2._ For cell passage, HUVECs were normally cultured in Ham’s F-12 K (PM150910, Procell, Wuhan) medium containing 10% FBS in an incubator at 37°C in 5% CO_2_.

### Cell transfection

The miR-934 inhibitor, miR-934 mimics, BTG2 siRNAs and their corresponding control were obtained from JTS scientific (Wuhan, China). They were transfected into indicated cells using Lipofectamine 2000 (11,668–019, Invitrogen, USA) according to the manufacturer’s instruction. Sequence information about miR-934 inhibitor, miR-934 mimics, BTG2 siRNAs and their corresponding control was listed in Table 1.

**Table 1.** siRNAs and related sequence information

Name	Sequence (5ʹ-3ʹ)
BTG2 siRNA-1 sense	CAGAGCACUACAAACACCATT
BTG2 siRNA-1 antisense	UGGUGUUUGUAGUGCUCUGTT
BTG2 siRNA-2 sense	GAGCAAGCAAGGUUAGCAATT
BTG2 siRNA-2 antisense	UUGCUAACCUUGCUUGCUCTT
BTG2 siRNA-3 sense	CUCAGUCACUGUGCAAUAUTT
BTG2 siRNA-3 antisense	AUAUUGCACAGUGACUGAGTT
hsa-miR934 mimics sense	UGUCUACUACUGGAGACACUGG
hsa-miR934 mimics antisense	AGUGUCUCCAGUAGUAGACAUU
NC mimics sense	UUCUCCGAACGUGUCACGUTT
NC mimics antisense	ACGUGACACGUUCGGAGAATT
hsa-miR934 inhibitor	CCAGUGUCUCCAGUAGUAGACA
NC Inhibitor	UUGUACUACACAAAAGUACUG

### RNA extraction and quantitative real-time PCR

Total RNA from CRC cells were extracted according to the protocol of RNA rapid extraction kit (RP1201, BioTeke Corporation). RNAs were then reversely transcribed into cDNAs by M-MLV reverse transcriptase (2641A, Takara). The mRNA levels of miR-934 and BTG2 were further quantified by quantitative real-time PCR using SYBR Green dye on ExicyclerTM96 machine (Bioneer Co, Daejeon, Korea). U6 was used as internal control gene for miRNA-934 quantification, while β-actin served as the control gene for BTG2. The relative expressions of them were calculated as 2^−ΔΔCT.^ Primers used in our study were synthesized by GenScript (Nanjing, China) and primer sequences were listed as follows:

Hsa-miR-934 F: 5ʹGTCTACTACTGGAGACACTGGG3ʹ;

Hsa-miR-934 R: 5ʹGCAGGGTCCGAGGTATTC3ʹ;

Hsa-U6 F: 5ʹGCTTCGGCAGCACATATACT3ʹ;

Hsa-U6 R: 5ʹGTGCAGGGTCCGAGGTATTC3ʹ;

BTG2 F: 5ʹCATCATCAGCAGGGTGGC3ʹ;

BTG2 R: 5ʹCCAATGCGGTAGGACACC3ʹ;

β-actin F: 5ʹGGCACCCAGCACAATGAA3ʹ

β-actin R: 5ʹ TAGAAGCATTTGCGGTGG3ʹ

### MTT assay

Cells were seeded in a 96-well plate at the density of 4 × 10^3^/well, and MTT assay was conducted according to the manufacturer’s protocol. Briefly, 0.5 mg/ml MTT (C0009, Beyotime Biotechnology) was added into each well at time point of 0, 24 h, 48 h and 72 h after transfection. Cells were incubated with MTT for 4.5 h at 37°C. The suspension was subsequently removed and cells were incubated with DMSO for another 10 min in the dark. The absorbance at 570 nm was recorded for analysis.

### Cell cycle analysis

Cells were seeded in a 6-well plate at the density of 4 × 10^5^/well, and cell cycle analysis was performed using Cell cycle detection kit (C1052, Beyotime Biotechnology) after transfection. Basically, cells were collected by centrifugation and then washed by pre-cooled phosphate-buffered saline (PBS) for three times. Subsequently, cells were fixed with pre-cooled 70% ethanol at 4°C overnight. After washing twice, cells were suspended by dyeing buffer (500 μL) and stained with another 25 μL of PI for 30 min in the dark at room temperature. The flow cytometry analysis was further performed for cell cycle determination (NovoCyte, ACEA Bioscience, USA).

### Western-blot analysis

Transfected cells were collected and then lysed by radioimmunoprecipitation assay (RIPA) buffer (P0013B, Beyotime Biotechnology) containing 1% phenylmethanesulfonyl fluoride (PMSF) (ST506, Beyotime Biotechnology). Total protein level of each sample was quantified by BCA quantization Kit (P0009, Beyotime Biotechnology). Equal amount of protein was loaded and separated on sodium dodecyl sulfate polyacrylamide gel electrophoresis (SDS-PAGE) gel, and then transferred onto polyvinylidene fluoride (PVDF) membrane for further analysis. After blocking, membranes were incubated with primary antibody anti-Ki67 (1:1000, A2094, Abclonal), anti-cyclin D1 (1:1000, #2922, CST), anti-MMP9 (1:1000, 10,375-2-AP, Proteintech), anti-MMP2 (1:500, 10,373-2-AP, Proteintech), anti-VEGF (1:1000, DF7470, Affinity), and anti-BTG2 (1:500, ab85051, Abcam) at 4^0^C overnight. After rinsing, membranes were incubated with horseradish peroxidase (HRP) -conjugated rabbit/mouse antibodies for 40 min at 37°C. Protein bands were visualized by enhanced chemiluminiscent (ECL) solution and analyzed by Gel-Pro-Analyzer (Media Cybernetics, Bethesda, MD, USA). The β-actin was used as an internal control.

### Wound Healing Assay

Transfected cells were cultured to 100% confluence, and the culture medium was changed into serum free medium supplemented with 1 μg/ml mitomycin C (M0503, Sigma, USA). A scratch was made to these cells by a 200 μl of pipette tip and cells were further cultured for 48 h. Images were obtained at 0 and 48 h after wounding under the microscope (IX53, OLYMPUS, Japan). The distance between edges was measured for analysis [20].

### Transwell assay

Transwell assay was performed to analyze cell invasive ability of CRC cells after transfection. Accordingly, transwell inserts (3422, Coming, USA) were placed in a 24-well plate and pre-coated with matrigel in advance (356,234, BD, USA). Subsequently, transfected cells were collected and seeded in the upper chamber of transwell insert. Cells were further cultured for 8 h at 37°C in a humidified incubator with 5% CO_2_ incubator. After blocking with 4% paraformaldehyde (C104188, Aladdin reagent, China), cells were stained with 0.4% crystal violet (0528, Amresco, USA) for 5 min. Representative images were captured under microscope (IX53, OLYMPUS, Japan) and invasive cells were calculated in five fields for further analysis [21].

### Enzyme-linked immunosorbent assay (ELISA)

The contents of VEGF from supernatant of transfected cells were determined by ELISA assay kit according to the manufacturer’s protocol (SEA143Hu, USCN Life Science, China).

### Tube formation assay

Pre-cooled matrigel (40 μL/well; 356,234, BD, USA) was added into a 96-well plate and incubated for 2 h at 37°C. HUVECs (CL-0122, Procell Life Science &Technology, Wuhan) were seeded in the plate coated with matrigel and cultured in tumor cell-conditional medium (TCM) for another 12 h. In details, TCM means the cell supernatant of transfected CRC cells. Representative photographs were subsequently captured under the microscope (IX53, OLYMPUS, Japan) after co-culture. Meanwhile, the branch numbers of associated tubes were counted [22], and normalized to the mean value of control group for the calculation of tube formation ratio.

### Bioinformatics database

In this study, bioinformatics database Starbase (ENCORI: The Encyclopedia of RNA Interactomes. (sysu.edu.cn)) was used to predict the potential association between miR-934 and BTG2 [23]. The function ‘miRNA-target’ in Starbase was employed to find the potential target genes of miR-934, and we finally selected BTG2 as the downstream gene of miR-934 for its anti-proliferative ability and aberrantly regulated expression in CRC [18].

### Dual luciferase reporter assay

293 T cells were seeded in a 12-well plate at 70% confluence, and they were starved by being incubated with serum free culture medium for 1 h. The co-transfection of miR-934 mimics/NC mimics and BTG2 wild type/BTG2 mutant was subsequently performed on 293 T cells using lipofectamine 2000 (11,668–019, Invitrogen, USA). Luciferase activities were quantified by the dual luciferase reporter assay kit according to the manufacture’s instruction (E1910, Promega, USA).

### Statistical Analysis

The statistic differences among groups were determined by one-way ANOVA followed by Tukey’s tests using GraphPad Prism. Data were presented as mean± standard deviation and p < 0.05 was considered as significant.

## Results

### The expression of miR-934 in colorectal cancer cell lines

**Figure 1.**
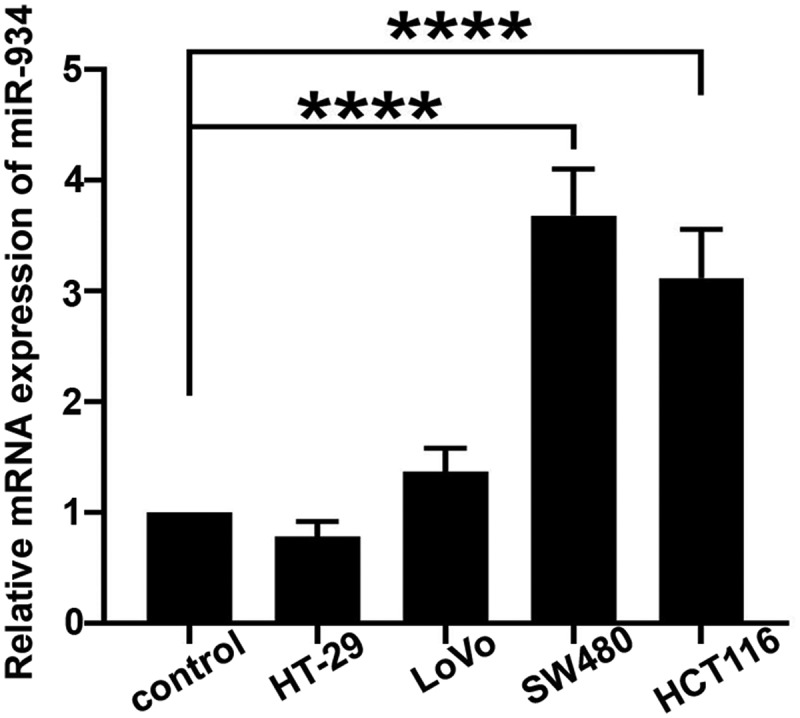
**The expression of miR-934 in colorectal cancer (CRC) cell lines**. Expression levels of miR-934 in colorectal cancer cell lines (HT-29, LoVo, SW480 and HCT116) and normal colonic epithelial cells were detected by qRT-PCR. Bars and error bars represent mean values and the corresponding SD, n = 3. *P < 0.05, **P < 0.01, ***P < 0.001, ****P < 0.0001

The real-time PCR was performed for the measurement of miR-934 mRNA level in CRC cell lines. Results in Figure 1 indicated that the expression of miR-934 was significantly increased in SW480 and HCT116 cells when compared to the normal cell line, and we finally chose SW480 and HCT116 cells for further study.

### MiR-934 deficiency inhibits cell proliferation and induces cell cycle arrest in colorectal cancer cell lines

**Figure 2.**
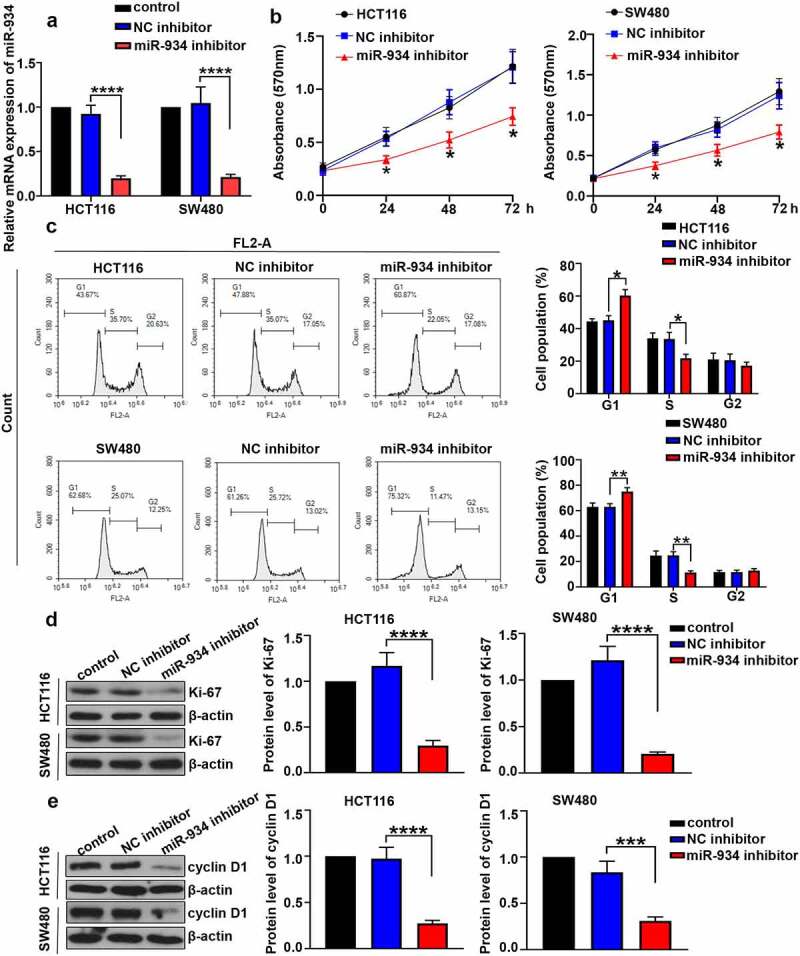
**The deficiency of miR-934 inhibited cell proliferation and induced cell cycle arrest in colorectal cancer cell lines**. MiR-934 inhibitor (100 pmol) or NC inhibitor (100 pmol) was transfected into HCT116 and SW480 cells. (a) The transfection efficiency was subsequently detected by qRT-PCR. (b) Cell viability was determined by MTT assay. (c) Flow cytometry was performed to determine cell cycle in CRC cell lines. (d&e) The protein levels of Ki-67 and cyclin D1 in CRC cell lines was analyzed by western-blot. Bars and error bars represent mean values and the corresponding SD, n = 3. *P < 0.05, **P < 0.01, ***P < 0.001, ****P < 0.0001

To investigate the effect of miR-934 on cell proliferation and cell cycle of CRC, miR-934 knockdown was respectively achieved in HCT116 and SW480 cells by the transfection of miR934 inhibitor. CCK-8 assay and cell cycle analysis were further performed after transfection. As indicated in Figure 2a, expression level of miR-934 in HCT116 and SW480 cells was significantly down-regulated after the transfection of miR-934 inhibitor. Besides, cell viability in HCT116 and SW480 cells was significantly suppressed after miR-934 knockdown (Figure 2b). Cell cycle analysis further indicated that miR-934 knockdown induced cell cycle arrest in G1 phrase in HCT116 and SW480 cells (Figure 2c). Moreover, the protein levels of Ki-67 and cyclinD1 in HCT116 and SW480 cells was determined to be significantly down-regulated after the knockdown of miR-934 (Figure 2d-e).

### Loss of miR-934 suppresses cell migration and cell invasion in colorectal cancer cell lines

**Figure 3.**
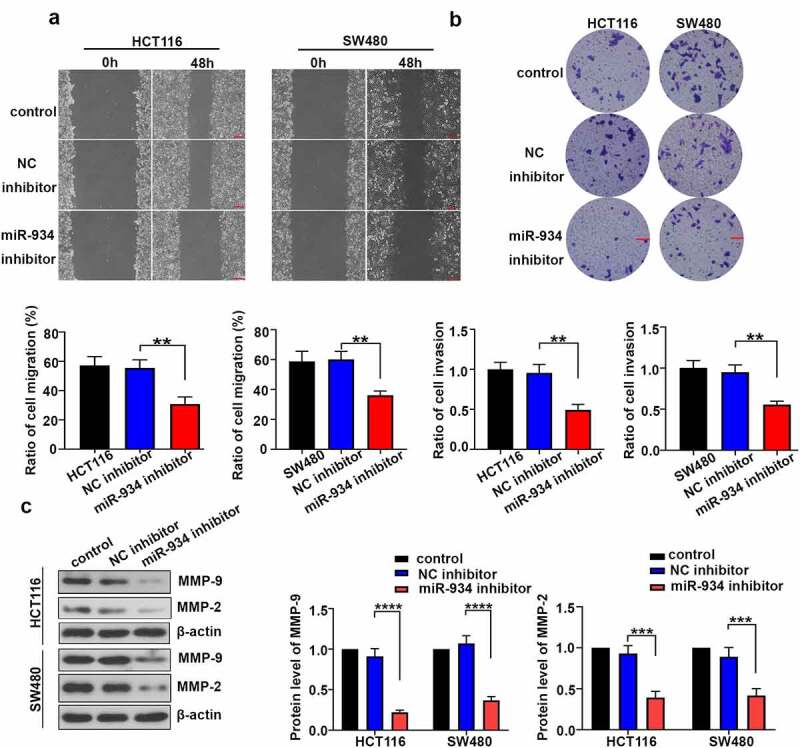
**Down-regulated miR-934 suppressed cell migration and invasion in colorectal cancer cell lines**. (a) Wound healing assay was performed to measure migration ability of HCT116 and SW480 cells after miR-934 knockdown. Scale bar: 200 μm. (b) Transwell assay was conducted to detect the invasive capacity of HCT116 and SW480 cell line after t after miR-934 knockdown. Scale bar: 100 μm. (c) The protein levels of MMP-9 and MMP-2 were respectively determined by western-blot analysis in HCT116 and SW480 cells after miR-934 knockdown. Bars and error bars represent mean values and the corresponding SD, n = 3. *P < 0.05, **P < 0.01, ***P < 0.001, ****P < 0.0001

To clarify the effect of miR-934 on cell migration and invasion of CRC cell lines, wound healing assay and transwell assay were respectively performed on HCT116 and SW480 cells. In general, cell migration rate was significantly inhibited in HCT116 and SW480 cells after miR-934 knockdown (Figure 3a). Additionally, miR-934 deficiency remarkably reduced cell invasion rate in HCT116 and SW480 cells (Figure 3b). The expression levels of MMP-9 and MMP-2 were further detected by western-blot analysis, and they were significantly down- regulated after miR-934 knockdown (Figure 3c).

### Knockdown of miR-934 restricts angiogenesis in colorectal cancer cell lines

**Figure 4.**
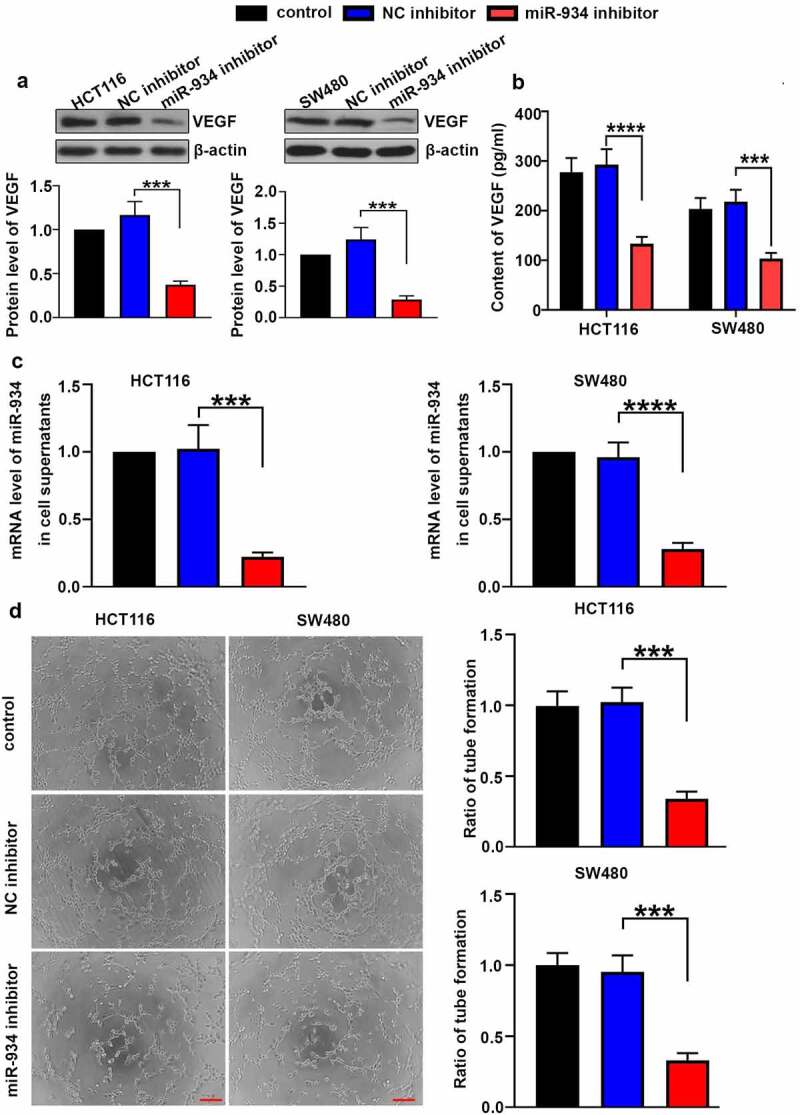
**miR934 knockdown depressed angiogenesis in colorectal cancer cell lines**. HCT116 and SW480 cells were transfected with miR-934 inhibitor (100 pmol) and NC inhibitor (100 pmol). After transfection, protein levels (a) and supernatant content (b) of VEGF were subsequently measured by western-blot and ELISA. (c) The mRNA levels of miR-934 in cell supernatants were determined by real-time PCR. (d) The representative images of tube formation in HUVECs were captured after the co-culture in TCM of SW480 and HCT116 cells, which was followed with calculation of tube formation ratio. Scale bar: 200 μm. Bars and error bars re present mean values and the corresponding SD, n = 3. *P < 0.05, **P < 0.01, ***P < 0.001, ****P < 0.0001

To validate the function of miR-934 in CRC angiogenesis, intracellular and extracellular VEGF was respectively detected by western-blot and ELISA after miR-934 knockdown. As shown in Figure 4a, the protein level of VEGF in HCT116 and SW480 cells were markedly reduced after the down-regulation of miR-934. Besides, results in ELISA indicated that VEGF content in cell supernatant was also significantly decreased after miR-934 knockdown (Figure 4b). Meanwhile, extracellular miR-934 was validated to be significantly down-regulated in HCT116 and SW480 cells after the transfection of miR-934 inhibitor (Figure 4c). Tube formation assay was further performed in vitro to explore the effect of miR-934 on tumor angiogenesis. We found that miR-934 knockdown suppressed capability of HCT116 and SW480 cells to promote tube formation in vascular endothelial cells (Figure 4d), suggesting that the deficiency of miR-934 could inhibit CRC angiogenesis.

### BTG2 is the direct target gene of miR-934 in colorectal cancer cell lines

**Figure 5.**
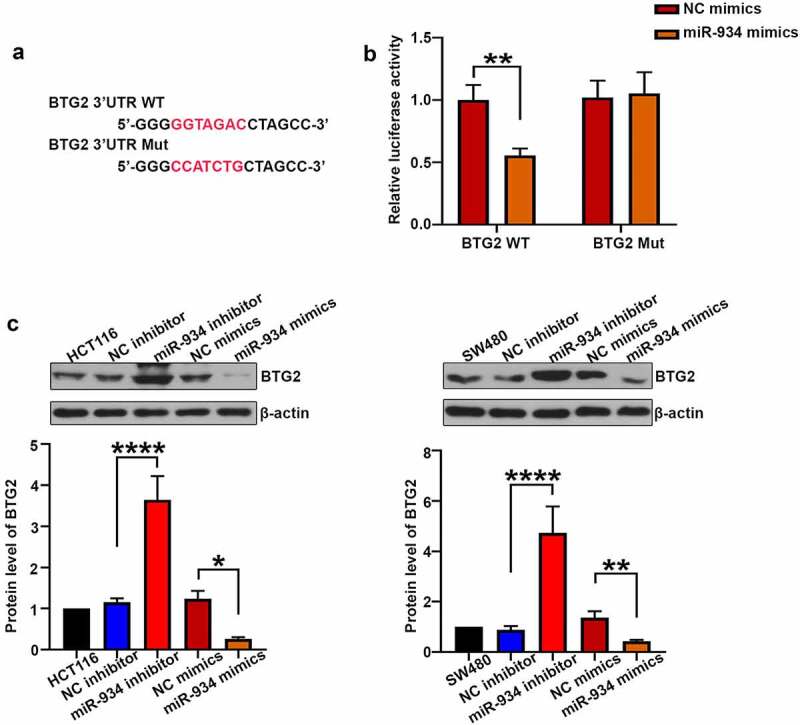
**BTG2 was a target of miR-934**. (a) The mutant and original site in 3ʹUTR of BTG2. (b) The dual luciferase reporter assay was performed to validate that BTG2 was the target gene of miR-934. (c) western-blot analysis was conducted to measure the protein level of BTG2 in HCT116 and SW480 cell line after transfection of miR-934 inhibitor (100 pmol), miR-934 mimics (100 pmol) and their negative control. Bars and error bars represent mean values and the corresponding SD, n = 3. *P < 0.05, **P < 0.01, ***P < 0.001, ****P < 0.0001

To determine whether BTG2 is the target gene of miR-934, the dual luciferase activity assay was further performed. As shown in Figure 5b, miR-934 mimics remarkably decreased luciferase activity of wild type BTG2 when compared with the NC mimics group, suggesting that miR-934 could bind with BTG2. Moreover, miR-934 knockdown and over-expression were respectively achieved by the transfection of miR-934 inhibitor and miR-934 mimics in HCT116 and SW480 cells. As shown in Figure 5c, the expression level of BTG2 was significantly increased after miR-934 knockdown, while over-expression of miR-934 conversely decreased BTG2 expression.

### MiR-934/BTG2 axis mediates cell proliferation, migration, invasion and angiogenesis of colorectal cancer cell lines

**Figure 6.**
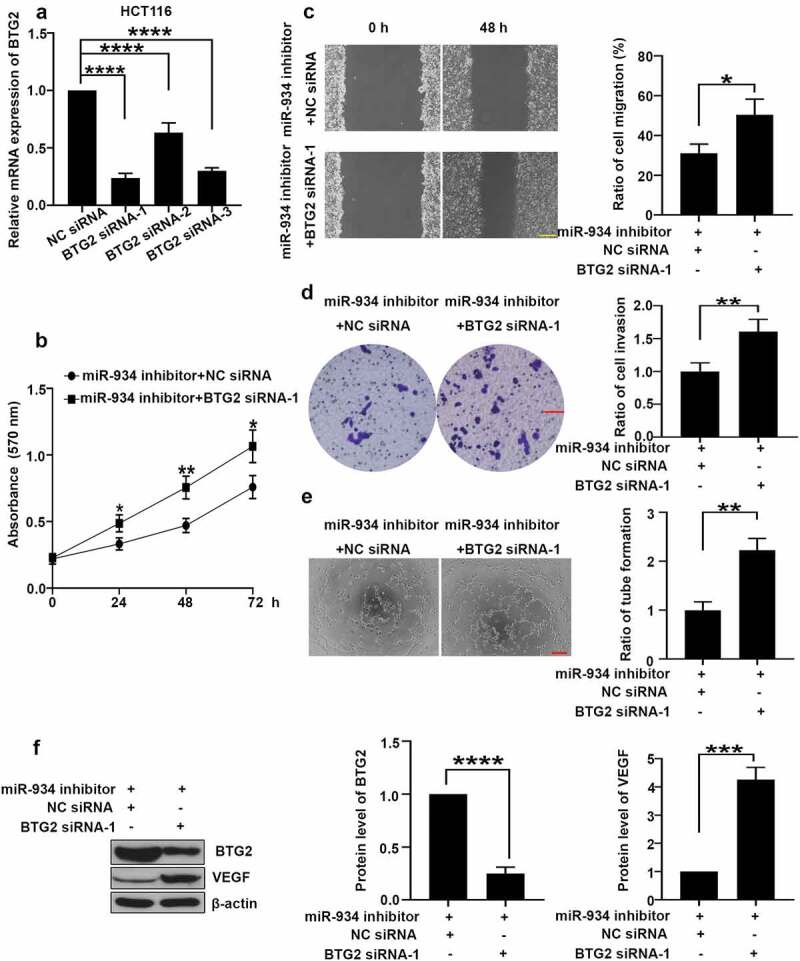
**miR-934 mediated cell proliferation, migration, invasion and angiogenesis in dependent with BTG2**. HCT116 cells were transfected with BTG2 siRNAs (50 pmol) and control siRNA with/without miR-934 inhibitor (50 pmol). (a) The mRNA levels of BTG2 in HCT116 cells were detected by qRT-PCR. (b) Cell proliferation was analyzed using MTT assay after the transfection. (c) The migratory ability of HCT116 cells was determined by wound healing assay. Scale bar: 200 μm. (d) The invasive ability of HCT116 cells was assessed by transwell assay. Scale bar: 100 μm. (e) The tube formation ratio of HUVECs were analyzed by tube formation assay. Scale bar: 200 μm. (f) The protein levels of BTG2 and VEGF were detected by western-blot. Bars and error bars represent mean values and the corresponding SD, n = 3. *P < 0.05, **P < 0.01, ***P < 0.001, ****P < 0.0001

To further investigate whether miR-934 mediates cell proliferation, migration, invasion and angiogenesis of CRC by targeting at BTG2, BTG2 knockdown was achieved by specific siRNA transfection in HCT116 cells (Figure 6a), and BTG2 siRNA-1 was selected for the following study. As shown in Figure 6b, BTG2 silencing remarkably promoted miR-934 knockdown-mediated cell proliferation in HCT116 cells. In comparison, analyses of wound healing and transwell assay indicated that BTG2 knockdown facilitated cell migration and invasion in HCT116 cells (Figure 6c-d) after miR-934 silencing. Furthermore, BTG2 knockdown reversed the inhibitory effect of miR-934 silencing on VEGF expression and tube formation in CRC cell lines (Figure 6e-f). In conclusion, miR-934 mediates cell proliferation, migration, invasion and angiogenesis in CRC by targeting at BTG2.

## Discussion

Colorectal cancer (CRC) is recognized as a leading cause of cancer–related deaths, making it a global public health concern. MicroRNAs (miRNAs) are a group of small non-coding RNAs that affect several biological processes by binding to target genes and inducing the degradation of target protein [24]. It should be noted that a great deal of aberrantly regulated miRNAs has been determined in CRC. Generally, they could serve as a suppressor or promoter of CRC and play an important part in regulating cell proliferation, migration, invasion and angiogenesis of CRC. For example, significantly reduced expression of miR-1258 is determined in CRC cells, and it is further verified to inhibit cell proliferation during CRC progression [25]. Besides, miR-532-5p is documented to promote aggressiveness of human CRC [26], further validating the essential roles of miRNAs in CRC.

As previously described, miR-934 plays an oncogenic role in a variety of cancers, and its expression is closely related to the pathological states. Pioneer researches have shown that highly expressed miR-934 is associated with poor prognosis in patients of pancreatic cancer and promotes cell proliferation in ovarian cancer [6,27]. Besides, exosomal miR-934 has been documented to promote liver metastasis of CRC by inducing M2 type macrophagy polarization [9], which validates the critical involvement of exosomal miR-934 in CRC progression. In this study, intracellular miR-934 was found to be over-expressed in SW480 and HCT116 cells rather than LoVo and HT-28 cells, which show higher expression of exosomal miR-934. We suppose that the source of miR-934 might be one reason for the difference in selecting typical cell line. Though previous researches have revealed part of relationship between miR-934 and CRC, the role of miR-934 in CRC pathogenesis has not been completely explored yet. This study further determined that miR-934 knockdown significantly inhibited cell proliferation, migration, invasion and angiogenesis in SW480 and HCT116 cells. Tumor cells are characterized with abnormal proliferative ability and enlarged life span beyond normal ones [28]. Effective interventions on cell proliferation have been regarded as a therapeutic strategy for malignancies [28,29]. Of note, cell cycle arrest acts as an inhibitory mechanism in tumor cell proliferation. In this study, miR-934 knockdown increased cell population in G1 phrase of CRC cells, validating the cell cycle arrest in these CRC cells. In addition, cyclin D1 expression was also decreased after miR-934 knockdown. Cyclin D1 plays a multifaceted role in tumor progression, while its major function is to transfer G1 phrase of cell cycle to S phrase, which induces DNA synthesis and promotes cell proliferation [30]. Additionally, promoted migratory and invasive capacity of tumor cells contributes to the process of cancer metastasis. In this study, miR-934 deficiency inhibited cell migration and invasion in CRC cell lines, validating that miR-934 could be a target for the management of cancer metastasis.

Tumor angiogenesis is mainly responsible for the supply of oxygen and nutrition to over-proliferative tumor cells, and it has been accepted as a rate-controlling step in CRC progression and metastasis [31]. More importantly, a diversity of angioregulatory miRNAs has been found to exert profound effects on CRC development. Accordingly, miR-181a facilitates angiogenesis of CRC via vascular endothelial growth factor (VEGF) signaling [13]. Tumor cells always facilitate angiogenesis by releasing a diversity of growth factors that induce formation of blood vessels, such as VEGF [32]. In our study, VEGF expression was markedly decreased after miR-934 knockdown in CRC cells, suggesting the inhibitory effect of miR-934 knockdown on angiogenesis of CRC. Besides, angiogenesis contributes to the formation of newly blood vessels [33], and the tube formation was found to be significantly restricted in HUVECs after miR-934 knockdown in CRC cells, further determining that miR-934 knockdown significantly inhibits tumor angiogenesis in CRC.

Increasing evidence has shown that miRNAs participate in gene regulatory network of various biological processes by binding to target mRNAs [34]. In our study, BTG2 was found to be the direct target of miR-934 and its expression was down-regulated in CRC cells after miR-934 over-expression. BTG2 has been identified as an anti-proliferative gene since 1991 [35,36], and it is recognized as a tumor suppresser in diverse malignancies [37,38]. Basically, BTG2 is an early response gene associated with cell cycle, DNA damage and tumor cell apoptosis [39]. BTG2 over-expression could lead to the down-regulation of cyclin D1 and induce cell cycle arrest in mouse embryo fibroblasts (NIH 3T3) [40]. Besides, over-expressed BTG2 also restricts cell migration and invasion in human renal carcinoma cell lines [41]. Moreover, miR-27a is determined to enhance angiogenesis in pancreatic cancer by targeting at BTG2 [19], which is concordant with our finding in CRC.

## Conclusions

In summary, our study indicates that the expression of miR-934 was significantly increased in SW480 and HCT116 cell lines. Besides, miR-934 knockdown significantly inhibited cell proliferation, migration, invasion and angiogenesis in CRC cell lines. More importantly, BTG2 was determined as the direct target of miR-934, and miR-934 facilitated cell proliferation, migration, invasion and angiogenesis in CRC by targeting BTG2.

## Data Availability

My manuscript has no associated data.
